# Density functional tight binding approach utilized to study X-ray-induced transitions in solid materials

**DOI:** 10.1038/s41598-022-04775-1

**Published:** 2022-01-28

**Authors:** Vladimir Lipp, Victor Tkachenko, Michal Stransky, Bálint Aradi, Thomas Frauenheim, Beata Ziaja

**Affiliations:** 1 Center for Free-Electron Laser Science CFEL, Deutsches Elektronen-Synchrotron DESY, Notkestr. 85, 22607 Hamburg, Germany; 2 European XFEL, 22869 Schenefeld, Germany; 3 Institute of Nuclear Physics, Polish Academy of Sciences, Radzikowskiego 152, 31-342 Kraków, Poland; 4 Institute of Physics of the Czech Academy of Sciences, Na Slovance 2, 182 21 Prague, Czech Republic; 5 Bremen Center for Computational Materials Science, Universitaet Bremen, Am Fallturm 1, 28359 Bremen, Germany; 6 Shenzhen JL Computational Science and Applied Research Institute, Shenzhen, 518110 China; 7 Beijing Computational Science Research Center, Beijing, 100193 China

**Keywords:** Materials science, Optics and photonics

## Abstract

Intense X-ray pulses from free-electron lasers can trigger ultrafast electronic, structural and magnetic transitions in solid materials, within a material volume which can be precisely shaped through adjustment of X-ray beam parameters. This opens unique prospects for material processing with X rays. However, any fundamental and applicational studies are in need of computational tools, able to predict material response to X-ray radiation. Here we present a dedicated computational approach developed to study X-ray induced transitions in a broad range of solid materials, including those of high chemical complexity. The latter becomes possible due to the implementation of the versatile density functional tight binding code DFTB+ to follow band structure evolution in irradiated materials. The outstanding performance of the implementation is demonstrated with a comparative study of XUV induced graphitization in diamond.

## Introduction

Recent advances in the development of free electron lasers (FEL) enable unprecedentedly precise observation of electron and nuclear dynamics in various samples^1,2^, giving rise to a number of important applications, including crystallographic structure determination^3–6^, investigation of valence- and core-electron dynamics^7–9^ and studies of ultrafast phase transitions^10^. At the same time, FELs enable new insights into the properties of warm dense matter and nanoplasmas^11–16^. Irradiation with X-rays can also trigger electronic, structural and magnetic transitions in solid materials, opening promising prospects for controlled processing of such materials.

The latter application is in the focus of this study which introduces a computational approach, enabling to study chemically complex materials irradiated with ultrashort intense X-ray pulses. Currently, in solid state physics, the majority of computational tools are based on Density Functional Theory (DFT)^17,18^ because of its accuracy and reasonable computational efficiency (see, e.g.^19,20^). However, if large-system (over 1000 particles) or long-timescale (longer than picoseconds) simulations are needed, the computational costs of a DFT-based approach become very high. In this case, semi-empirical methods, such as Tight Binding (TB)^21,22^ or Density Functional Tight Binding (DFTB)^23–26^ provide a reasonable precision at a much lower computational cost. In case of X-ray irradiation, additional obstacle is high energy of X-ray excited electrons (up to keV). Such range of electronic energies is challenging to be treated with a fully ab-initio method^27^. With this in mind, our in-house simulation tool XTANT (X-ray-induced Thermal And Non-Thermal transitions)^28,29^ has been developed. It represents a computationally efficient hybrid approach which allows to study X-ray and XUV irradiated solids. The main principle behind the construction of XTANT is the separation of electrons into low and high energy fractions. This is justified by the specific shape of the transient electron distribution, which is typically observed upon X-ray irradiation of solids. It includes contribution of thermalized electrons at low energy range, and the so called ‘bump on hot tail’ contribution of non-thermalized electrons (originating from photoinduced processes) at high energy range^11,30^. Relaxation of high energy electrons and core-hole excitation are followed with the Monte Carlo (MC) approach, which takes into account photoionization and electron impact ionization, modeled with appropriate cross sections^29^. The decay of core holes is also described by the MC module, which accounts for finite core hole lifetimes and their complex relaxation paths. Band electrons from low-energy electron fraction, which is separated from high-energy electron fraction by a cutoff energy of $\sim$ 10 eV^31^, are treated quantum mechanically. They are assumed to always obey thermal Fermi-Dirac distribution.

The response of the band structure to any changes of nuclei positions is modeled with the transferable tight binding (TTB) approach (e.g.,^21^). Atomic motion is traced using classical molecular dynamics (MD), with forces calculated within the TTB module. More detailed description of XTANT modules can be found in the Supplementary Information.

The simulation tool, XTANT can provide transient thermodynamic^32,33^ and optical parameters^10,34^ as well as atomic trajectories within irradiated samples. Its reliability has been successfully validated against a number of experimental studies^10,34,35^. Notably, in Ref.^10^, the comparison of the experimental and theoretical optical property, transient transmissivity, allowed to identify temporal timescales for various stages of X-ray induced ultrafast graphitization of diamond.

However, the XTANT code relies on the empirical TTB approach for band structure calculations. It is, therefore, not versatile, as simulations of various materials require that the respective TTB parametrizations are manually introduced to the main code. Also, the TTB approach becomes less reliable at states far from equilibrium. For example, the simulations of X-ray-induced warm dense matter (WDM)^36^ yielded only a qualitative agreement with the experimental predictions^16^.

In this study, we present an implementation of the well known open-source code DFTB+^26^ into our code XTANT. The DFTB+ code will be used to perform band structure calculations in response to changes of nuclei positions and electronic temperature, triggered by photoinduced and collisional processes in X-ray irradiated solid materials. Since the DFTB+ approach is both more rigorous and more versatile than the phenomenological TTB parametrization, this implementation will significantly extend the limits of the applicability of the XTANT code, offering new capabilities such as a wide choice of targets and possibility to include the spin effects. Still, the newly extended code, called from now on XTANT+, preserves a reasonable computational efficiency, allowing long-timescale and large-scale simulations of X-ray irradiated materials (see ‘Supplementary Information’). The proposed scheme of connecting the DFTB+ and XTANT codes, overcoming the problem of differing thermodynamic ensembles for electronic subsystems used in these codes, can be used in any future implementations of the DFTB+ code.

## Results

As we discussed above, the TTB parameterization in the band structure calculation module of the XTANT code can be replaced with any other model that can calculate electron levels, $\left\{ \epsilon _i \right\}$ as a function of the nuclei positions. It is, however, essential that $\left\{ \epsilon _i \right\}$ only depend on the nuclei position and not on other parameters such as, e.g., the electronic temperature. This assumption holds with a good accuracy for the case of the versatile DFTB+ code^26^. To justify it, we performed test calculations of the band structure with the original DFTB+ code. For two electronic temperatures, 300 K and 30,000 K, the relative differences in the positions of electronic energy levels were less than 0.3%.

In the ‘Methods’ section we present details of the force calculation in the XTANT and DFTB+ codes, as well as the new algorithm allowing to utilize DFTB+ as a band structure calculation module of XTANT+ (for more details on the modular structure of XTANT+ see the Supplementary Information). The algorithm overcomes the problem of the different thermodynamic ensembles used for electronic subsystems in these codes. The idea is to separate the calculation of forces and energy levels from the calculation of the electronic occupations (redistributed on the newly calculated energy levels) by introducing additional computational substeps. This ensures the desired conservation of the total energy (not of the Mermin free energy). This step is essential, as it enables accurate control of the energy absorption during the interaction of an irradiated sample with X rays^37^.

With this implementation, one can study various (also chemically complex) materials, which band structure has been already parametrized, or can be relatively easy calculated with the DFTB+ tool. Below we show the first application of XTANT+ to study response of diamond to intense X-ray pulses. The diamond has been extensively studied by us with the original XTANT code, e.g.,^28,29,32,36^. In what follows, we will show and compare the predictions obtained with XTANT and XTANT+ codes.

### Band gap evolution in diamond as a function of absorbed dose

As a first step, we performed a set of simulations with the following X-ray pulse parameters: 10 fs duration, 50 eV photon energy and various values of the X-ray dose absorbed in the bulk diamond, ranging from 0.2 eV/atom up to 2.5 eV/atom. The results were obtained with the total number of 216 atoms in the supercell which in this case yields sufficiently accurate predictions, according to the convergence study presented in the ‘Supplementary Information’. In addition, each predicted curve was averaged over 7 MD realizations.

**Figure 1:**
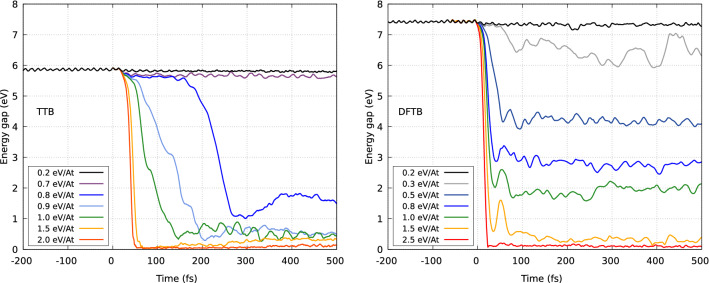
Evolution of band gap in XUV-irradiated diamond predicted with (left) the original XTANT approach, and with (right) XTANT+. The XUV pulse had a duration of 10 fs, the photon energy was 50 eV. The calculations were performed using a 216-atom supercell. The curves represent averages over 7 MD realizations. The figure has been generated using gnuplot 4.6 patchlevel 4 (http://gnuplot.info).

Figure 1 presents the evolution of the band gap at different absorbed doses, predicted with XTANT (using the TTB method for band structure calculation) and with XTANT+ (using DFTB+ for band structure calculation). While for the XTANT model both the magnitude and the timescale of the band gap decrease strongly depend on the absorbed dose, in XTANT+ it is mainly the magnitude, which changes for the absorbed doses above 0.3 eV/atom. Also, the timescale of the band gap decrease is similar at the absorbed doses above 0.5 eV/atom. However, the magnitudes of the band gap decrease expected with XTANT and XTANT+ differ for the same dose. This is due to the differing band gaps predicted for ambient diamond with these codes (6.0 eV vs. 7.5 eV). As the band gap in ambient diamond with XTANT+ is wider than in XTANT, higher doses are needed in this case to initiate a similar decrease of the band gap width as observed with XTANT.

The observed discrepancy in the temporal behaviour of band gap decrease predicted with two code versions cannot be easily explained. Experimental verification would help to validate the modeling accuracy. Such measurement could be possible with a pump probe scheme, such as, e.g., X-ray pump—optical probe scheme discussed in^38^.

Note that in the earlier XTANT simulations^28^, the damage threshold in diamond was found to be about 0.7 eV/atom. Here, after identifying and removing an inconsistency in the energy reference level within the TTB-based band structure module, the XTANT predicts the dose $\sim$ 1 eV/atom (as Fig. 1 shows). The new code, XTANT+, predicts the full closure of the band gap at the dose of above 1.5 eV/atom. This can be again explained by the discrepancy in the band gap width for ambient diamond which in XTANT+ is by $\sim$ 1.3 eV larger than in XTANT.

### Graphitization of diamond predicted with XTANT+

**Figure 2:**
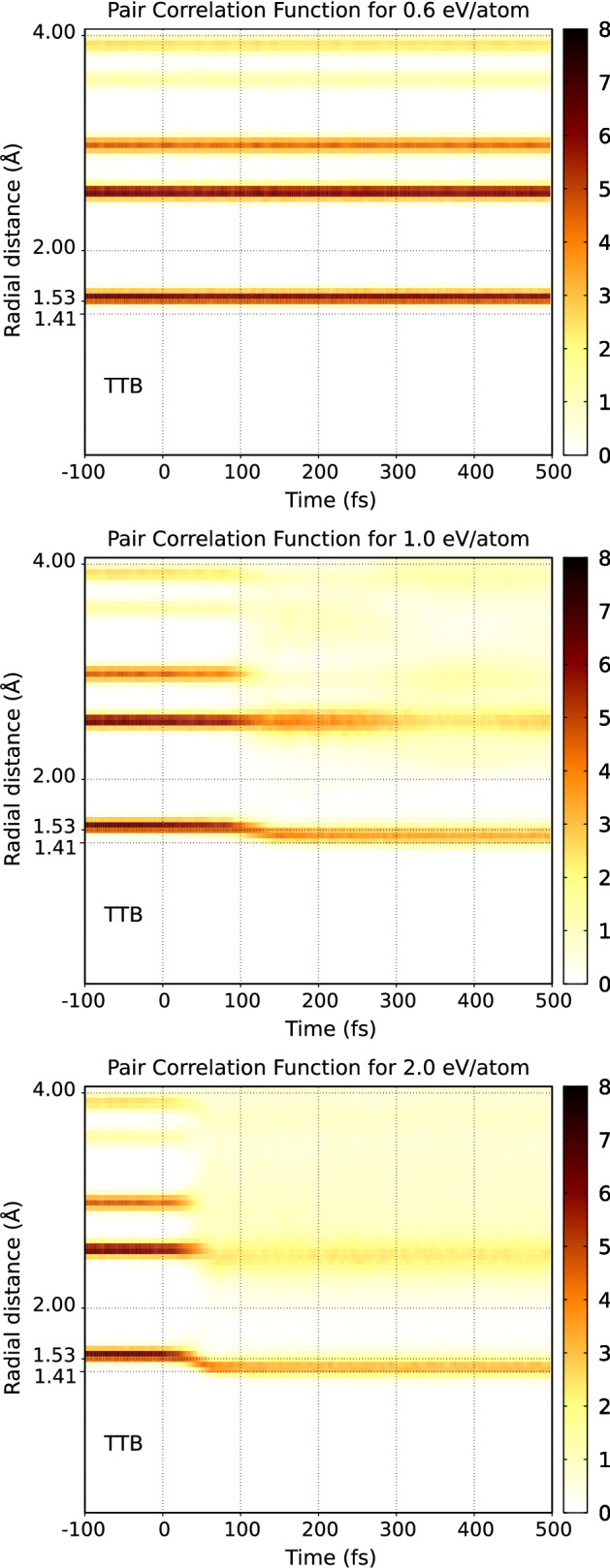
Temporal evolution of pair correlation function for various X-ray absorbed doses: (top) 0.6 eV/atom, (center) 1.0 eV/atom, and (bottom) 2.0 eV/atom in diamond bulk as predicted by **XTANT**. Pulse parameters are the same as in Ref.^10^: XUV photon energy of 47.4 eV, pulse duration 52.5 fs FWHM. The calculations were performed with a 512-atom supercell. The figure has been generated using gnuplot 5.0 patchlevel 6 (http://gnuplot.info).

**Figure 3:**
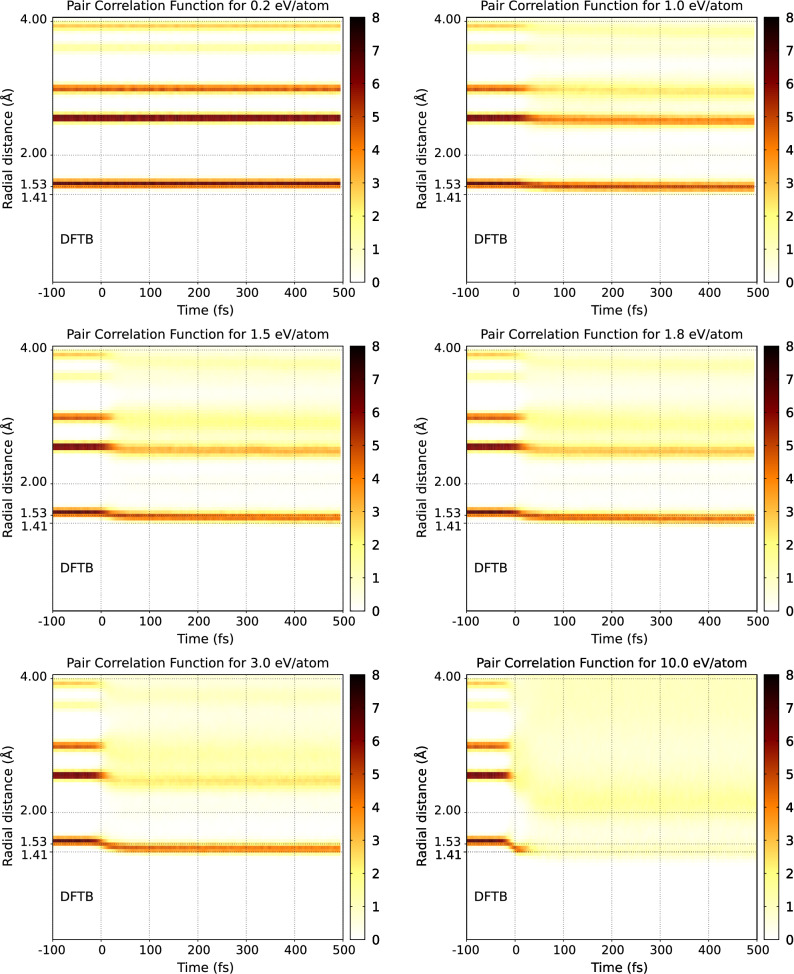
Temporal evolution of pair correlation function for various X-ray absorbed doses in diamond bulk as predicted by **XTANT+**. Pulse and supercell parameters are the same as in Fig. 2. The figure has been generated using gnuplot 5.0 patchlevel 6 (http://gnuplot.info).

As we already mentioned above, one of the successful applications of the original XTANT code was the simulation of the XUV-induced graphitization of diamond^10^. Below we demonstrate that XTANT+ can also reasonably follow this process. The progress of the structural transition can be conveniently traced with the transient (spherically averaged) pair correlation function (PCF), sensitive to the changes of material structure^28^. Figures 2 and 3 show the evolution of the PCF predicted with the XTANT and XTANT+ codes respectively. As discussed above, we know that in the TTB simulations ultrafast graphitization occurs at the dose $\sim$ 1 eV/atom. However, since the band gap predicted by the DFTB+ is noticeably larger ($\sim$ 7.5 eV), while in TTB it is $\sim$ 6 eV, one can expect that a higher dose is needed to trigger such transition in XTANT+.

**Figure 4:**
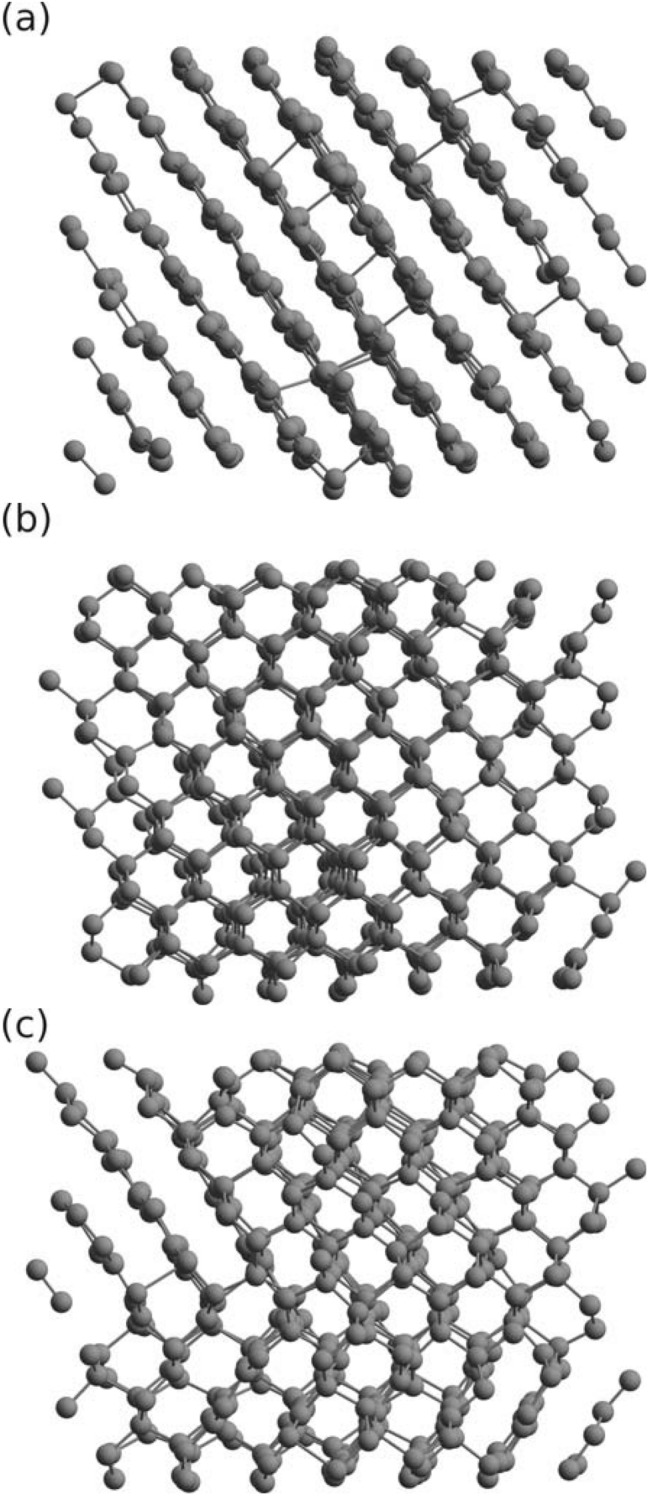
Snapshots of graphitization and partial graphitization occurring in X-ray irradiated diamond obtained with (**a**) XTANT at dose 1.0 eV/atom, (**b**) XTANT+ at dose 1.3 eV/atom, and (**c**) XTANT+ at dose 2.0 eV/atom. Figures show 3D view of the atomic structure in the supercell at $t=500$ fs. Pulse and supercell parameters are the same as in Fig. 2. The figure has been generated using Avogadro 1.2.0 (https://avogadro.cc)^39,40^.

Figure 2 shows the PCF obtained with XTANT for the doses of 0.6, 1.0, and 2.0 eV/atom respectively. Our earlier studies^28^ showed that graphitization leads to the shift of the first PCF peak from $\sim$ 1.53 to $\sim$ 1.41 Å. For the absorbed dose of 0.6 eV/atom, the structure of diamond remains stable during the whole simulation. For the absorbed dose of 1.0 eV/atom, the position of the first peak in PCF shifts to $\sim$ 1.41 Å. At the same time, the position of the second peak at $\sim$ 2.5 Å remains almost unchanged. This indicates the graphitization of diamond, as the attached snapshot at 500 fs confirms (Fig. 4a). At even higher dose of 2.0 eV/atom, all peaks smear out, which indicates a loss of order and a rapidly progressing amorphization of the sample.

Similar behaviour can be observed for the PCF predicted with the XTANT+ (Fig. 3). At the absorbed doses less than $\sim$ 1 eV/atom, the positions of PCF peaks do not change. At doses higher than $\sim$ 1.3 eV/atom, structural transitions occur: (i) with partial graphitization observed at the doses of $\sim$ 1.3–2.0 eV/atom (see the snapshots at 500 fs in Fig. 4b,c), and (ii) with amorphization at higher doses. In Fig. 3, the cases for the dose of 3.0 eV/atom and 10 eV/atom are shown. Note that with the increasing absorbed dose, the amorphization affects a larger number of PCF peaks. Also, both with XTANT and XTANT+, we obtain the same trend on the lowest peak: the larger the absorbed dose is, the lower the lowest PCF peak shifts.

It is interesting that graphitization predicted with XTANT+ results is a partial (‘broken’) graphitization occurring locally in two perpendicular planes. This is in contrast to the purely ‘planar’ graphitization predicted with XTANT which forms well ordered graphite planes. An NPH ensemble simulation, excluding the $V=const$ restriction, could help to understand better this discrepancy. However, the extension of the current XTANT+ scheme to enable NPH ensemble simulations is non-trivial, and, therefore, beyond the scope of the current study.

In experiments, X-ray pulses irradiating diamond samples are spatially non-uniform. They deposit different doses in various parts of the diamond sample. We expect that diamond cannot be converted fully into graphite upon absorbing a moderate X-ray dose. Therefore, partial nano-graphite structures should typically appear in crystal, as the XTANT+ simulation indicates. In Ref.^31^, such partial graphitization of diamond was also demonstrated with XTANT for a moderate absorbed dose.

## Discussion

We presented a versatile and consistent implementation of the DFTB+ code into the code XTANT. The updated code is now called XTANT+. A comparative study of XUV induced graphitization in diamond performed with two different approaches to calculate band structure: the TTB approach (in XTANT) and the DFTB+ scheme (in XTANT+) demonstrates the outstanding performance of the new implementation.

The electron-ion coupling is not taken into account in the current version of XTANT+. Based on the results of our previous work with XTANT code^41^, we expect that the electron-ion coupling should not significantly affect the sample dynamics on timescales relevant for graphitization of diamond (100–200 fs), as including or not including electron-ion coupling did not affect the non-thermal graphitization thresholds for diamond (Ref.^41^, Table I therein). However, this may be not the case on yet longer timescales. In order to investigate this, a dedicated study with XTANT+ (extended so as to include electron-ion coupling) is needed.

Let us also comment on the cutoff energy separating the low- and the high-energy electron fractions. The cutoff dependence was studied earlier with XTANT code^31^. Variations of the cutoff around 10 eV did not noticeably affect the simulation results. This observation should also hold, if we replace the TTB parametrization of band structure with the DFTB parametrization, as the value of the cutoff also corresponds to the uppermost conduction band states described by the DFTB method.

XTANT+ opens up unique opportunities to study X-ray-induced transitions in a variety of solid materials (or nanoobjects), with applications to diverse areas of research such as materials science, structural biology, crystallography, and fundamental physics. The evolution of the irradiated target material can be followed with optical coefficients^42^ and diffractive imaging^29^. Since the DFTB+ code is used in the XTANT+ unmodified, the implementation benefits from a fully consistent approach to describe evolution of band structure. This is in contrast to the application of the empirical TTB model in the original XTANT code. All existing DFTB+ extensions become now available for use in the context of X-ray irradiation. For example, one could include the electron spin effects or benefit from the GPU parallelization. Implementing the electron-phonon coupling in XTANT+ is not a part of this work, but it can be relatively straightforward accounted for, in a manner similar as in the original XTANT code^41^. Also, an extension of the current scheme to the NPH ensemble is possible. The proposed scheme of connecting two codes, which implement differing thermodynamic ensembles for electronic subsystems, can also be utilized in future applications of DFTB+.

## Methods

### Force calculation in XTANT and DFTB+ computational tools

Linking of DFTB+ to the XTANT code enables a straightforward calculation of the energy levels in the electronic band, and of the forces acting on the nuclei, upon providing actual coordinates of the nuclei, $\mathbf {R}=\{R_k\}$ (where index *k* runs over all nuclei in the system) and the actual electronic temperature, $T_e$, by the XTANT code. However, special care must be taken about thermodynamic ensemble for the electronic subsystem.

The DFTB+ code (similar to the majority of ab initio DFT codes) determines the occupations $f_i = f(\epsilon _i, \mu _\mathrm {e}, T_\mathrm {e})$ of one-electron eigenstates, $\{ \epsilon _i \}$ through the Fermi-Dirac distribution function, *f*. The distribution function depends on the electronic temperature $T_\mathrm {e}$, and on the chemical potential of the electrons $\mu _\mathrm {e}$. The latter is determined automatically by ensuring the conservation of the total number of electrons:

1 $$\begin{aligned} N_\mathrm {e} = \sum _i f_i . \end{aligned}$$

Thermodynamically, this procedure corresponds to an NVT ensemble, where the number of electrons, the volume of the system and the temperature of the electrons are kept fixed during the simulation.

The DFTB total energy up to a second order can be written as:

2 $$\begin{aligned} E_\mathrm {DFTB} = E_\mathrm {band} - \frac{1}{2} \sum _{AB} \gamma _{AB} (q_B - q_B^0) (q_A + q_A^0) + E_\mathrm {rep} , \end{aligned}$$

where $E_\mathrm {band}$ is the energy of band electrons, $E_\mathrm {rep}$ is the repulsive energy, $\gamma _{AB}$ is the Coulomb-like coupling between the gross charges on atoms *A* and *B*. The quantity $q_A$ denotes the Mulliken electron population of atom *A*, and $q_A^0$ is the electron population of a reference (neutral) atom of the same element^26,43^. The summation goes over all atoms present in the sample (see the Supplementary Information).

The band energy can be calculated as:

3 $$\begin{aligned} E_\mathrm {band} = \sum _i f_i \, \epsilon _i . \end{aligned}$$

Upon a change of the nuclei positions, the gradient of the band energy becomes:

4 $$\begin{aligned} \nabla _{\mathbf {R}}\, E_\mathrm {band}=\nabla _{\mathbf {R}}\, \sum _i f_i \, \epsilon _i=\sum _i (\nabla _{\mathbf {R}}f_i \cdot \epsilon _i) +\sum _i (f_i \cdot \nabla _{\mathbf {R}}\epsilon _i). \end{aligned}$$

containing the gradient of the occupation numbers as well as the gradient of one-electron levels. However, when considering an NVT ensemble for the electrons, it is not the band energy, but the Mermin free energy, $\tilde{E}_\mathrm {band} = E_\mathrm {band} - T_e S$, which represents the correct thermodynamical potential. Here, *S* denotes the electronic entropy. If electronic occupations follow the Fermi-Dirac statistics, the gradient of the entropy term $T_e S$ equals to the gradient of the occupations $f_i$ in Eq. (4)^44^, so that the gradient of the Mermin free energy can be reduced to:

5 $$\begin{aligned} \nabla _{\mathbf {R}}\, \tilde{E}_\mathrm {band} = \nabla _{\mathbf {R}}\, \left( E_\mathrm {band} - T_e S \right) = \sum _i (f_i \cdot \nabla _{\mathbf {R}}\epsilon _i) . \end{aligned}$$

Consequently, when carrying out the NVE Born-Oppenheimer nuclei dynamics (by not attaching any heat reservoir to the ions and by bringing the electrons to their ground state at $T_e$ at every time step), it is the energy:

6 $$\begin{aligned} \tilde{E} = E_\mathrm {DFTB} - T_e S + E_\mathrm {kin} = \tilde{E}_\mathrm {DFTB} + E_\mathrm {kin} = const , \end{aligned}$$

which will be conserved during the dynamics. The energy $E_\mathrm {kin}$ denotes the kinetic energy of the ions. This results in an ‘impure’ NVE dynamics, where the electrons (but not the ions) are coupled to a heat reservoir. Consequently, not the total energy, $E = E_\mathrm {DFTB} + E_\mathrm {kin}$ but the electronic-entropy corrected energy, $\tilde{E} = E - T_e S$ is conserved.

In contrast to the DFTB, the XTANT scheme uses a “pure” NVE thermodynamic ensemble for both the electron and ion dynamics, without applying a thermostat for electrons. This enables to accurately control the energy absorbed in the interaction of irradiated sample with X-rays. The implementation of NVE ensemble in XTANT follows the scheme introduced for the first time in^37^.

XTANT scheme assumes that electronic Fermi-Dirac occupations, $f_i = f(\epsilon _i, T_e,\mu _e)$ do not explicitly depend on the nuclei positions. Therefore, in Eq. (4) one can neglect the first term, since $\nabla _{\mathbf {R}}f_i =0$. As a result, one obtains the same analytical expression for the gradient of the band energy as in Eq. (5), $\nabla _{\mathbf {R}}\,E_\mathrm {band} = \sum _i (f_i \cdot \nabla _{\mathbf {R}}\epsilon _i)$, however, under different assumption. Therefore, in XTANT the total energy:

7 $$\begin{aligned} E = E_\mathrm {DFTB} + E_\mathrm {kin} = const , \end{aligned}$$

is conserved during the dynamics. With this in mind, we could propose a rigorous scheme to link DFTB+ to XTANT. The details can be found in the next subsection.

### Algorithm allowing to utilize DFTB+ as a module of XTANT

Below we show how to link the DFTB+ band structure calculation module to XTANT, in a way consistent within the NVE scheme used by XTANT. Within a single time step, the following substeps should be performed:

1. Input from XTANT on the initial conditions, i.e., actual nuclei coordinates, $\mathbf {R}=\{R_k\}$, actual number of band electrons, $N_e$, and actual electronic temperature $T_e$ is provided to the DFTB+ code serving as a band structure calculation module.
2. With this data, DFTB+ performs a respective self-consistent charge (scc-DFTB) calculation. It yields: (i) the actual electronic energy levels $\left\{ \epsilon _i \right\}$, (ii) the actual Fermi-Dirac electronic occupations, $\left\{ f_i = f(\epsilon _i, T_e,\mu _e)\right\}$, with $\mu _e$ calculated, using the known $T_e$ and $N_e$, (iii) and forces. Note that the forces used by DFTB+ are calculated similarly to XTANT by assuming that the electronic Fermi-Dirac occupations, $f_i = f(\epsilon _i, T_e,\mu _e)$, do not explicitly depend on the nuclei positions (see Eq. (5)). Therefore, they can be directly used in the XTANT algorithm. The band energy at this step is: $E_\mathrm {band} = \sum _i f(\epsilon _i, T_e,\mu _e) \, \epsilon _i$, see Eq. (3).
3. XTANT lets the forces to act on nuclei, moving them to new positions, $\mathbf {R}=\{R_k\} \rightarrow \mathbf {R}^{\prime }=\{R_k^{\prime }\}$. Data on $\{R_k^{\prime }\}$ are transferred to the DFTB+ code.
4. Another scc-DFTB calculation with the new atomic positions is performed, in order to obtain new energy levels $\left\{ \epsilon ^\prime _i\right\}$. Force calculation is switched off at this substep.
5. Consistently with the scheme applied in the XTANT, the electronic occupations, $\left\{ f_i = f(\epsilon _i, T_e,\mu _e)\right\}$, do not change during nuclei relocation. The corresponding new band energy is, therefore, defined as: $E^\prime _\mathrm {band} = \sum _i f_i \epsilon ^\prime _i = \sum _i \, f(\epsilon ^\prime _i, T_e,\mu _e) \, \epsilon ^\prime _i\,$.
6. The electrons are then redistributed on the new energy levels, $\left\{ \epsilon ^{\prime }_i\right\}$, keeping their total band energy equal to $E^\prime _\mathrm {band}$. The new Fermi-Dirac electronic occupations, $f_i^\prime = f(\epsilon _i^\prime , T_e^{\prime },\mu _e^{\prime })$ have to fulfill the condition, $E^\prime _\mathrm {band} = \sum _i \, f_i^\prime \epsilon ^\prime _i$. The energy conservation condition and the electron number conservation enable to calculate new values of $T_e^{\prime }$ and $\mu ^\prime _e$, as prescribed by the following constraints: $N_e = \sum _i f^\prime _i = \sum _i f(\epsilon ^\prime , T^\prime _e,\mu ^\prime _e)$ and $E^\prime _\mathrm {band} = \sum _i f^\prime _i \epsilon ^\prime _i = \sum _i f(\epsilon _i^\prime , T^\prime _e,\mu ^\prime _e) \epsilon ^\prime _i$. Note that in this substep we have applied the assumption that a change of electronic temperature does not affect the energy levels. This condition holds exactly for non-scc DFTB and with a reasonable accuracy for scc-DFTB (see Supplementary Information for details) .
7. In the final substep, DFTB+ calculates the forces which will then act on the nuclei at the next time step. For this, the updated electronic temperature, $T_e^{\prime }$, the actual number of band electrons, and the actual nuclear positions are used. The algorithm then effectively returns to the substep 3, and later repeats the subsequent substeps.
8. The procedure is repeated, until the calculation time is finished.

A dynamic simulation carried out with the scheme described above keeps the total energy *E* of the system conserved, leading to a “pure” NVE dynamics for both electrons and atoms. It should be noted, that although in the current scheme two DFTB calculations are carried out at each time step, only one DFTB calculation per time step would be necessary, if the search for the electronic temperature were implemented directly within the band structure calculation module (and not performed externally in XTANT). We plan to extend DFTB+ in the near future with that feature, and expect the simulation efficiency to be significantly improved afterwards.

The corresponding Slater-Koster parametrization files utilized in the DFTB+ module can be found in Refs.^45–47^. In order to validate the DFTB+ implementation, we performed convergence studies for the differing time steps and the system size (see ‘Supplementary Information’ for details). The test simulations showed that the time step of 50 as ensures reliable energy and particle number conservation. In addition, we confirmed that simulations with a supercell containing 216 atoms are reasonably similar to those containing 512 atoms. In ‘Supplementary Information’ we also show the behaviour of various physical parameters predicted by the new scheme for an application example, XUV irradiated diamond.

## Supplementary Information

Supplementary Information.
