# Supplementary material for: Density functional tight binding approach utilized to study X-ray-induced transitions in solid materials
Source: Sci Rep. 2022 Jan 28;12:1551. doi: 10.1038/s41598-022-04775-1 (PMC8799736; doi:10.1038/s41598-022-04775-1)
Supplement: Supplementary file 1 — Supplementary Information. [file 41598_2022_4775_MOESM1_ESM.pdf]

Supplementary Information:
Density Functional Tight Binding approach utilized to study X-ray-induced transitions in solid materials

Vladimir Lipp,[1] Victor Tkachenko,[2,3,4] Michal Stransky,[2,5]
Bálint Aradi,[6] Thomas Frauenheim,[6,7,8] and Beata Ziaja[9,3]

[1]*Center for Free-Electron Laser Science CFEL,
Deutsches Elektronen-Synchrotron DESY,
Notkestr. 85, 22607 Hamburg, Germany.
E-mail: vladimir.p.lipp@desy.de*

[2]*European XFEL, Holzkoppel 4, 22869 Schenefeld, Germany*

[3]*Institute of Nuclear Physics, Polish Academy of Sciences,
Radzikowskiego 152, 31-342 Kraków, Poland*

[4]*Center for Free-Electron Laser Science CFEL,
Deutsches Elektronen-Synchrotron DESY,
Notkestr. 85, 22607 Hamburg, Germany*

[5]*Institute of Physics of the Czech Academy of Sciences,
Na Slovance 2, 182 21 Prague, Czech Republic*

[6]*Bremen Center for Computational Materials Science,
Universitaet Bremen, Am Fallturm 1, 28359 Bremen, Germany*

[7]*Shenzhen JL Computational Science and Applied
Research Institute, Shenzhen 518110, China*

[8]*Beijing Computational Science Research Center, Beijing 100193, China*

[9]*Center for Free-Electron Laser Science CFEL,
Deutsches Elektronen-Synchrotron DESY,
Notkestr. 85, 22607 Hamburg, Germany.
E-mail: ziaja@mail.desy.de*

(Dated: November 19, 2021)

I. MODULES OF CODES, XTANT AND XTANT +

For the simulations of X-ray induced damage processes in diamond, we applied our hybrid codes XTANT (X-ray-induced Thermal And Nonthermal Transitions [1, 2]) and its updated version, XTANT+, introduced in the main paper. The codes have been developed specifically for studying response of solid materials to ultrafast X-ray irradiation. They include the the interconnected modules, described below. More details on each individual module of the code, their interconnections and the details of the numerical algorithm can be found in Ref. [2].

(a) Monte Carlo (MC) model which traces: X-ray photoabsorption, Auger-decays of core holes, and electron impact ionization. Photoabsorption cross sections, as well as any required rates and parameters, are extracted from the EPDL97 database [3] and from Refs. [4, 5]. For the electron impact ionization, the cross sections are obtained with the complex dielectric function approach [6]. Each photo-, Auger or collisionally emitted electron is traced until its kinetic energy falls below a certain cut-off energy (here set to 10 eV). Electrons with energies below the cutoff energy form source terms for the kinetic equations describing populations of low-energy electrons in the valence and conduction bands.

(b) Rate equations are used to trace the distribution of low-energy electrons. The equations describe electron populations on energy levels within the valence and conduction bands. The actual energy levels are calculated with the band structure calculation module (see (d)). Electron-electron scattering is assumed to instantaneously thermalize the electronic system so that the distribution function always adhere to a Fermi-Dirac one.

(c) Atomic motion is traced within a molecular dynamics (MD) framework, using Verlet algorithm. Atomic potential energy surface is calculated within the band structure calculation module (see (d)). Optionally, the energy provided via the nonadiabatic coupling of atoms to low-energy electrons (see (e)) can be introduced into the atomic system at each time-step via the velocity scaling.

(d) Band structure calculation module

In **XTANT**, the transferable tight binding (TTB) method is used to calculate the atomic potential energy surface in the solid. It also provides the corresponding forces, electronic band structure, and wave functions necessary for the evaluation of matrix elements entering Boltzmann collision integrals (see (e)), and the calculation of the complex dielectric function

within the random phase approximation [7]. For carbon, the transferable tight binding parameterization from Ref. [8] is used. Its applicability to electronically excited systems was confirmed for various systems and conditions such as diamond, graphite, amorphous carbon, C60 (for details see the review [2] and references therein). Changes in the atomic potential energy upon electronic excitation are naturally included within the TTB parametrization, thereby allowing us to model possible nonthermal effects, such as nonthermal melting and phase transitions, including ablation [2].

In **XTANT+**, the original DFTB+ code is linked as the band structure module. It performs band structure calculations on-the-fly.

(e) Boltzmann collision integral (BCI) describes nonadiabatic interactions between the electrons and atoms using the 'dynamical coupling' approach developed in [9]. It calculates the probability of an electron transition between two energy levels, triggered by atomic displacements. Note that this approach does not rely on the accuracy of the phononic approximation for the atomic motion (periodic lattice, harmonic vibrations, etc.). Instead, it is applicable to any kind of atomic motion and atom configuration.

Let us emphasize that, as our current studies are restricted to the timescales $<$ 500 fs, we do not account for the nonadiabatic electron-ion coupling here.

II. COMPUTATIONAL EFFICIENCY OF XTANT AND XTANT+

In order to compare the computational efficiency of the new model, we performed two test simulations, one with the XTANT code and another with the XTANT+ code. The parameters of the simulations correspond to those in Figs. 2 and 3 of the main manuscript: 600 fs runtime, 512-atom supercell with periodic boundary conditions, 50 eV photon energy, 10 fs pulse duration, and the absorbed dose of 1 eV/atom. The XTANT simulation has been completed within $\sim$ 2 days, whereas the corresponding XTANT+ runtime was $\sim$ 12 days, both on Intel Xeon CPU E5-2630L v4 at 1.80GHz using 4 cores. We can conclude that, although XTANT+ has a higher computational cost, it still enables reasonably efficient (long-timescale and large-spatial-scale) simulations.

III. CONSERVATION OF TOTAL ELECTRON NUMBER AS A FUNCTION OF SIMULATION TIMESTEP

Here we study the conservation of the total number of electrons as a function of simulation timestep. In the corresponding simulation, an equilibrated supercell consisting of 64 diamond atoms with periodic boundary conditions was irradiated with an XUV pulse of 10 fs duration; photon energy was 50 eV and the absorbed dose was 3 eV/atom. An XUV pulse (of temporal Gaussian shape with the maximum at time $t = 0$) excited high-energy electrons from band levels. In XTANT or XTANT+ models, such high energy electrons are removed from the band structure module and treated with the Monte Carlo module (as explained in the main manuscript). The total number of electrons obtained after adding up the number of the band electrons and the number of the high-energy electrons should always be conserved. Supplementary Figure 1 demonstrates the error in the electroneutrality, i.e., the difference between the total number of electrons and the initial number of electrons in valence band divided by the initial number of electrons in the valence band. The curves show that the error in the electroneutrality is negligible at all times, even for the largest studied timestep, 200 as. We conclude that the conservation of the total number of electrons is fulfilled at least for time steps of 200 as or shorter.

IV. ENERGY CONSERVATION AS A FUNCTION OF SIMULATION TIMESTEP

We also studied the accuracy of energy conservation as a function of the simulation time step. We again used a diamond supercell consisting of 64 atoms, with periodic boundary conditions imposed. In the simulations, the sample was equilibrated during the first 300 fs. Afterwards, its electronic temperature was instantaneously increased to 24000 K (t=0 in Supplementary Figure 2) which resulted in an absorbed dose of $\sim$ 0.7 eV/atom. That increase triggered a partial ultrafast band gap collapse, similarly as it was demonstrated in the main manuscript (Fig. 1 therein). The evolution of total energy of the supercell was then followed for different time steps: 20 as, 50 as, and 200 as. Supplementary Figure 2 presents the results. The curves are shown from the time instant at which the electronic temperature was increased. As expected, the smaller the time step, the better energy conservation. Even for 200 as time step, the energy drift is less than 10 meV/ps, which is much smaller when

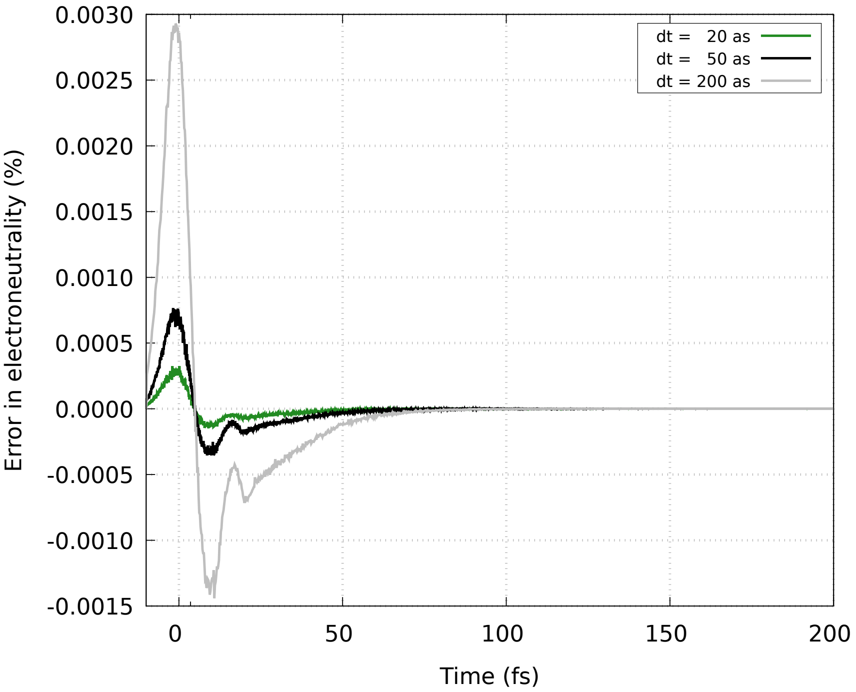

FIG. S1: Conservation of total electron number in simulations of XUV irradiated diamond performed with XTANT+. In the simulation, the XUV pulse had a duration of 10 fs, the photon energy was 50 eV, and the absorbed dose was 3.0 eV/atom. The figure has been generated using gnuplot 5.2 patchlevel 6 (http://gnuplot.info).

compared with the energy pumped into the system. Based on the predictions, we chose 50 as-long time step to perform simulations in the main manuscript, and in the section V. We additionally checked that for the maximal dose studied in this work (10 eV/atom), the chosen time step results in the energy drift of about 20 meV/ps.

V. CONVERGENCE OF XTANT+ WITH RESPECT TO SUPERCELL SIZE

Here, we studied the convergence of physical parameters of the simulated sample with respect to the size of the supercell. The simulation was performed under the same conditions as those in sec. IV. The electronic temperature was instantly increased from 300 K to 24000 K at time $t = 0$, triggering strong changes in thermodynamic parameters. In Supplementary Figure 3, the results obtained from simulations with 64-atom supercell, with 216-atom supercell, and 512-atom supercell simulations are shown. The comparison between different supercell sizes indicates that, although the 64 atom supercell is certainly too small

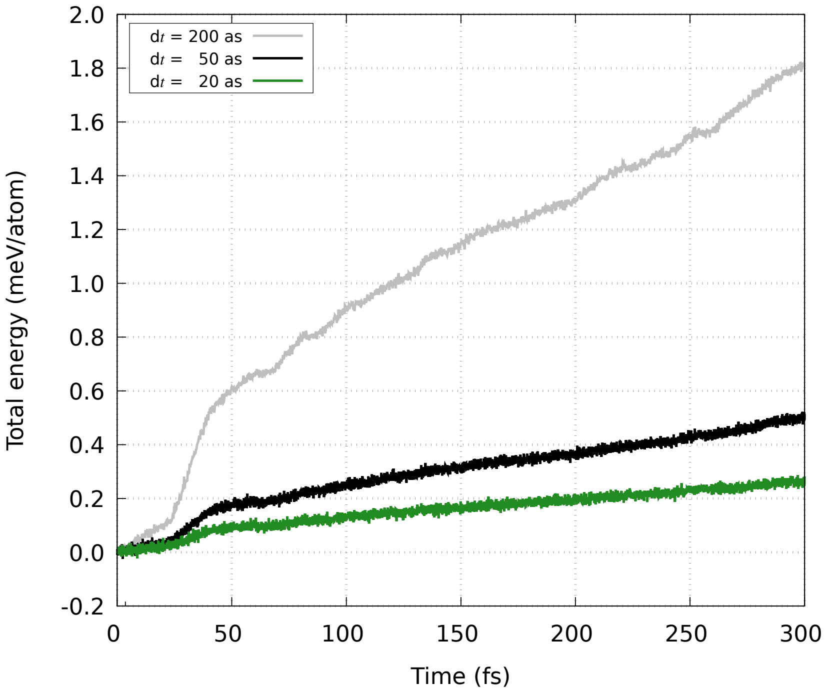

FIG. S2: Energy conservation in XTANT+ simulation. First, 64-atom diamond supercell is equilibrated at room temperature conditions. Afterwards, the electronic temperature is instantly increased to 24000 K. The curves show the subsequent evolution of the total energy for different time steps. The figure has been generated using gnuplot 5.2 patchlevel 6 (http://gnuplot.info).

to obtain converged results, the 216-atom supercell already provides converged parameters of the system. Therefore, we use 216- and 512-atom supercells in the main manuscript.

VI. DFTB APPROACH VERSUS TTB APPROACH

DFTB approach is derived from Kohn-Sham density functional theory by expanding the total energy functional [10]. The ground state density is represented by a reference density, ρ_0, and its perturbation by density fluctuations: $\rho(\vec{r}) = \rho_0(\vec{r}) + \delta\rho(\vec{r})$. The DFTB total energy is then written as a Taylor series to the second order :

$$E[\rho_0 + \delta\rho] = E^0[\rho_0] + E^1[\rho_0 + \delta\rho] + E^2[\rho_0, (\delta\rho)^2], \quad (1)$$

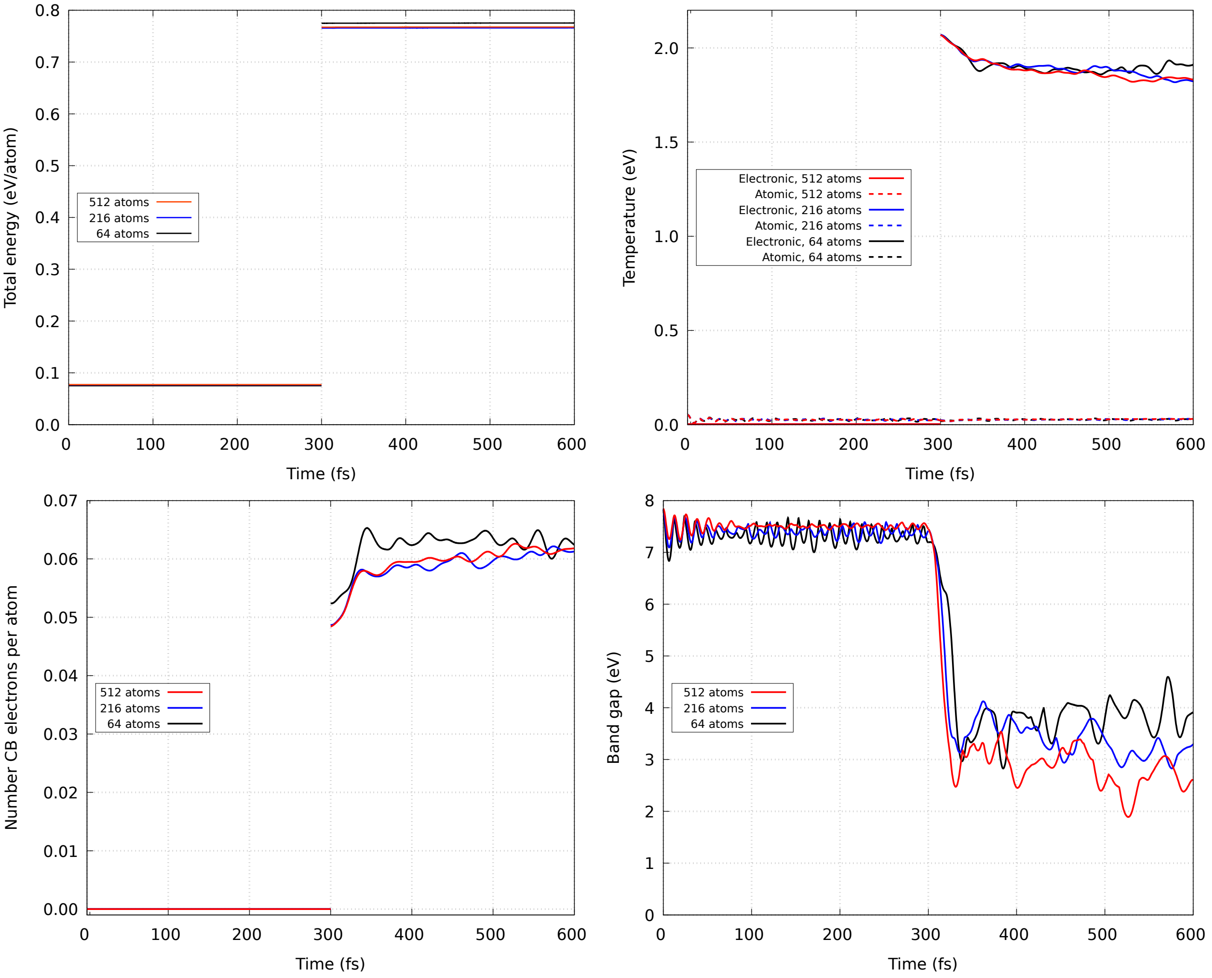

FIG. S3: Evolution of total energy (left top), temperatures (right top), electron density (left bottom), and energy gap (right bottom), calculated with XTANT+. The 50-as time step and various supercell sizes were used. Other parameters of the simulation are the same as in Supplementary Figure 2. The figures have been generated using gnuplot 5.2 patchlevel 6 (http://gnuplot.info).

where

$$\begin{aligned} E^0[\rho_0] =& \frac{1}{2}\sum_{AB}\frac{Z_A Z_B}{R_{AB}} - \frac{1}{2}\int\int \frac{\rho_0(\vec{r})\rho_0(\vec{r'})}{\left|\vec{r}-\vec{r'}\right|}\mathrm{d}\vec{r}\mathrm{d}\vec{r'} \\ & - \int V^{\mathrm{XC}}[\rho_0]\rho_0(\vec{r})\mathrm{d}\vec{r} + E^{\mathrm{XC}}[\rho_0], \\ E^1[\rho_0+\delta\rho] =& Tr\left(\rho\hat{H}[\rho_0]\right), \\ E^2[\rho_0,(\delta\rho)^2] =& \frac{1}{2}\int\int\left\{\frac{1}{\left|\vec{r}-\vec{r'}\right|} + \left.\frac{\delta^2 E^{\mathrm{XC}}}{\delta\rho(\vec{r})\delta\rho\vec{r'}}\right|_{\rho_0}\right\}\delta\rho(\vec{r})\delta\rho(\vec{r'})\mathrm{d}\vec{r}\mathrm{d}\vec{r'} \end{aligned} \tag{2}$$

with E^{XC} and V^{XC} being the exchange correlation energy and potential respectively. They are typically fitted from *ab initio* calculations. In the context of DFTB, $E^0[\rho_0]$ is sometimes called "repulsive energy". Recently, the third-order expansion of the total energy has also been implemented [10].

The total energy, Eq. (1), can be rewritten as follows:

$$E[\rho_0 + \delta\rho] = E^0[\rho_0] + \sum_i f_i \epsilon_i - \frac{1}{2} \sum_{AB} \gamma_{AB}(q_A + q_A^0)(q_B - q_B^0), \tag{3}$$

where ϵ_i are the electronic levels, f_i are the corresponding electronic occupations, and q_{X} is the actual charge of atom X (q_{X}^0 is the reference charge of neutral atom). In this form, the energy is no longer written as a Taylor expansion (except of the first term), since both the second and the third term depend on the second-order terms.

In case of the non-scc DFTB, the last term in Eq. (3) is zero and $Tr\left(\rho \hat{H}[\rho_0]\right) = \sum_i f_i \epsilon_i$, making the approach equivalent to a transferable tight binding approximation.

The second-order scc-DFTB contributions typically become significant for ionic materials, biological materials, or materials consisting of more than one element. In such cases, however, only the contribution to the total energy is expected to be significant, whereas the band structure can usually be described reliably with the non-scc-DFTB. For diamond, the non-scc-DFTB should work accurately in most cases. However, here we showed that a scc-DFTB based description of diamond is also possible. In particular, we demonstrated the consistent implementation of the full model, paving the way for future applications for more complex materials.

Let us emphasize that the present model is based on the assumption that electronic levels do not implicitly depend on the electronic temperature. This assumption holds accurately for the non-scc-DFTB. In the scc-DFTB, it is only true approximately, since the second-order scc term (the last term in Eq. (3)) contains f_i via $q_X = \hat{S}\rho$, where $\hat{S}$ is the overlap matrix. However, the precise energy conservation achieved in this study indicates that the approximation is accurate enough.

Based on the actual model, it should be possible to extend the DFTB+ code, including fully consistent NVE ensemble for both atomic and electronic subsystems. Currently, only atoms obey a NVE ensemble, whereas a thermostat is applied for electrons which then represent a NVT ensemble. Such extension would probably require an additional self-correcting loop to adjust the electronic energies at every time step. The resulting extended model

would then not require the ϵ_i to be independent of the electronic temperature, and should be reliable even if second-order contributions are essential.

VII. XTANT CODE APPLIED TO SIMULATE IONIZATION DYNAMICS WITHIN X-RAY IRRADIATED DIAMOND

Supplementary Figure 4 presents the evolution of diamond irradiated with a 10-fs-long XUV pulse, calculated with the original XTANT code. Photon energy was 50 eV, and the absorbed dose was equal to 0.6 eV/atom. The simulations were performed for 512-atom supercell, with periodic boundary conditions. The lattice parameter was 3.56683 Å. The left upper plot in Supplementary Figure 4 shows evolution of various energies of the target: atomic kinetic energy, E_{kin}, atomic potential energy, $E_e + E_{rep}$, and the total energy of the system, $E_{kin} + E_e + E_{rep}$. The latter is conserved before and after the laser action, whereas in-between it increases by the amount of the absorbed energy, 0.6 eV/atom. This confirms that the energy conservation is fulfilled and that the model has been correctly implemented. At the top right panel of Supplementary Figure 4, one can see the evolution of electronic and atomic temperatures. The electron-phonon coupling is not taken into account in this simulation. Therefore, all the absorbed energy remains confined in the electronic subsystem which temperature then stays almost constant. The left bottom panel shows the density of the band electrons with energies below 10 eV (green line) and high-energy Monte-Carlo electrons (blue line). The latter exist only during the laser pulse, because the electron cascading time is very short for 50 eV incident photons. Finally, the plot on the bottom right presents the evolution of the chemical potential (blue line) and the band gap (red line). The latter is initially ~ 6 eV, which corresponds to the case of undamaged diamond in the TTB approximation. It does not change noticeably during the simulation indicating the lack of damage. Chemical potential shows a characteristic step-like increase early in the exposure, following the increase of the electronic temperature. Afterwards, it remains almost constant, as expected.

VIII. XTANT+ CODE APPLIED TO SIMULATE IONIZATION DYNAMICS OF X-RAY IRRADIATED DIAMOND

Supplementary Figure 5 shows the application of XTANT+ to simulate evolution of X-ray irradiated diamond under the same irradiation conditions as in Supplementary Figure 4. The total energy (Eq. (7) in the main manuscript) is conserved before and after the X-ray pulse, confirming the correctness of the implementation of NVE ensemble. Up to our knowledge, the DFTB approach was not reported to be used with this ensemble before. In general, all curves in Supplementary Figure 5 look reasonably similar to those in Supplementary

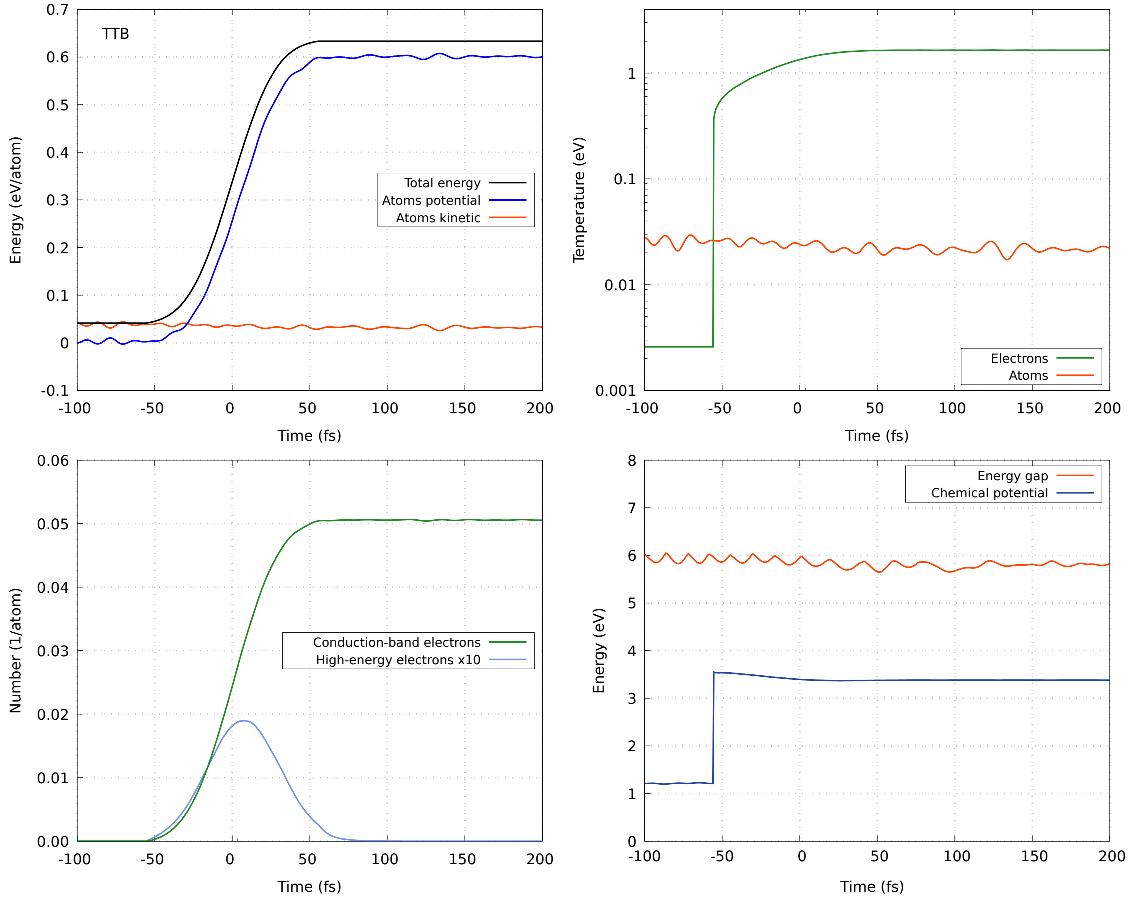

FIG. S4: Evolution of XUV-irradiated diamond predicted using the original XTANT approach with transferable tight binding parametrization. The XUV pulse had a duration of 10 fs, the photon energy was 50 eV, and the absorbed dose was 0.6 eV/atom. The supercell contained 512 atoms. The figures have been generated using gnuplot 5.2 patchlevel 6 (http://gnuplot.info).

Figure 4. A notable difference is the value of the equilibrium band gap, which is $\sim$ 7.5 eV in the DFTB model, compared to $\sim$ 6 eV in the TTB case. Another difference is that the band gap slightly decreases at the dose of 0.6 eV/atom, unlike for the TTB case, consequently increasing the number of excited electrons after the laser action is already finished.

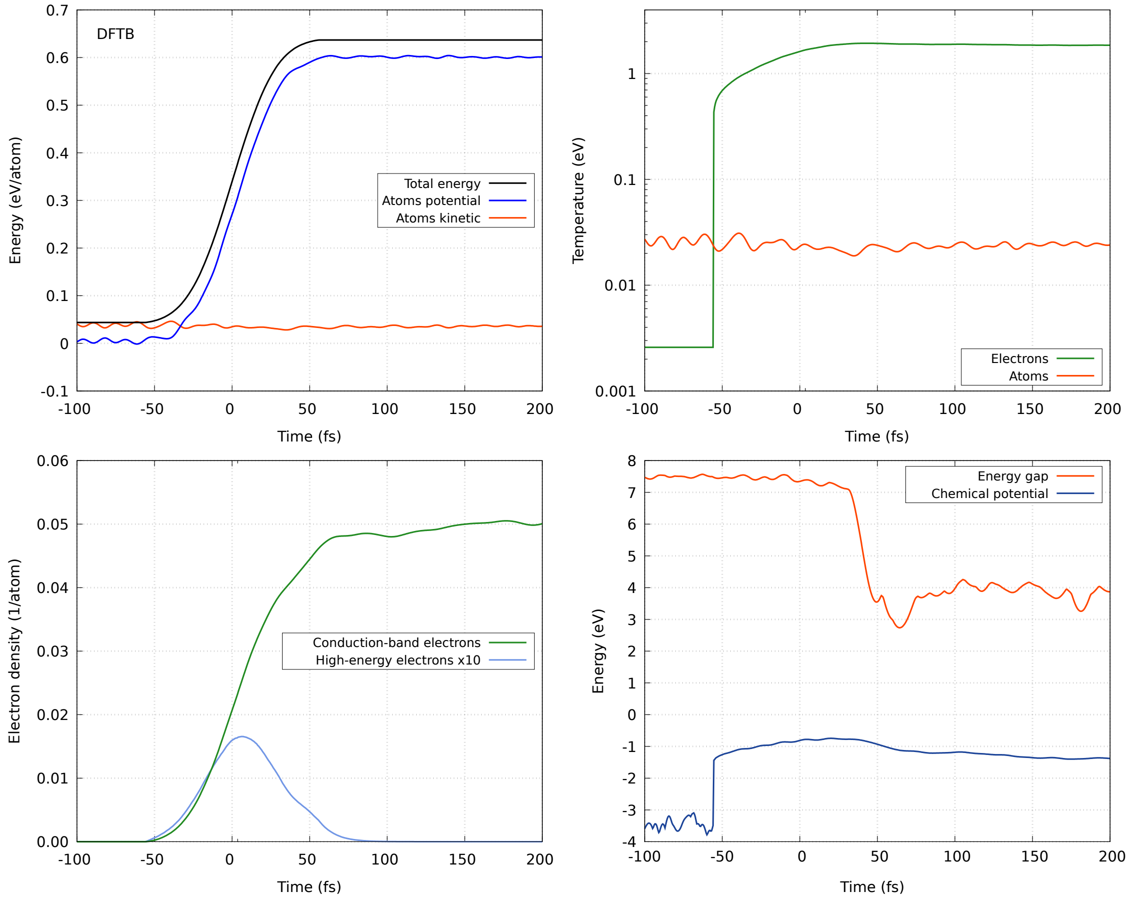

FIG. S5: Evolution of XUV-irradiated diamond studied using the new XTANT+ approach with the DFTB+ module implemented. The simulation parameters are the same as in Supplementary Figure 4. The figures have been generated using gnuplot 5.2 patchlevel 6 (http://gnuplot.info).

[1] Medvedev, N., Jeschke, H. & Ziaja, B. Nonthermal phase transitions in semiconductors induced by a femtosecond extreme ultraviolet laser pulse. *New J. Phys.* **15**, 015016 (2013).

[2] Medvedev, N., Tkachenko, V., Lipp, V., Li, Z. & Ziaja, B. Various damage mechanisms in carbon and silicon materials under femtosecond x-ray irradiation. *4open* **1**, 3 (2018).

[3] Cullen, D. E. et al. EPDL97: the Evaluated Photon Data Library,'97 version. *Lawrence Livermore National Laboratory, Livermore, CA* **6** (1997).

[4] Palik, E. D. Handbook of Optical Constants of Solids. *Academic Press handbook series, Academic Press, San Diego.* **1** (1985).

[5] Perkins, S. T. Tables and Graphs of Atomic Subshell and Relaxation Data Derived from the LLNL Evaluated Atomic Data Library (EADL). Z = 1-100. *Lawrence Livermore National Laboratory, Livermore, CA* **30** (1991).

[6] Akkerman, A. et al. Inelastic Electron interactions in the Energy Range 50 eV to 10 keV in insulators: Alkali Halides and Metal Oxides. *Phys. Stat. Sol. B* **198**, 769 (1996).

[7] Tkachenko, V., Medvedev, N., Li, Z., Piekarz, P. & Ziaja, B. Transient optical properties of semiconductors under femtosecond x-ray irradiation. *Phys. Rev. B* **93**, 144101 (2016).

[8] Xu, C., Wang, C., Chan, C. & Ho, K. A transferable tight-binding potential for carbon. *J. Phys. Cond. Mat.* **4**, 6047–54 (1992).

[9] Medvedev, N., Li, Z., Tkachenko, V. & Ziaja, B. Electron-ion coupling in semiconductors beyond Fermi's golden rule. *Phys. Rev. B* **95**, 014309 (2017).

[10] Hourahine, B. *et al.* DFTB+, a software package for efficient approximate density functional theory based atomistic simulations. *J. Chem. Phys.* **152**, 124101 (2020).
